# Impact and Outcomes of Regional Anesthesia Techniques in Elderly Patients With Fracture of Proximal Femur: A Retrospective Study

**DOI:** 10.7759/cureus.19392

**Published:** 2021-11-09

**Authors:** Sandeep Diwan, André Van Zundert, Abhijit Nair, Parag K Sancheti, Chetan Pradhan, Chetan Puram

**Affiliations:** 1 Anaesthesiology, Sancheti Institute for Orthopaedics and Rehabilitation, Pune, IND; 2 Anaesthesia and Perioperative Medicine, Royal Brisbane and Women’s Hospital, Brisbane, AUS; 3 Anesthesiology, Ibra Hospital, Ibra, OMN; 4 Orthopaedics, Sancheti Institution for Orthopaedics and Rehabilitation, Pune, IND; 5 Orthopaedics, Sancheti Institute for Orthopaedics and rehabilitation, Pune, IND

**Keywords:** regional anesthesia, geriatric, fracture, femur, lumbosacral plexus, epidural anesthesia, spinal anesthesia

## Abstract

Background

Although subarachnoid block (SAB) is the most popular regional anesthesia (RA) technique for fixation of femur fractures, continuous lumbar epidural (CLE) anesthesia, and lumbosacral plexus blocks (LSPB) are also employed in specific situations. The choice of RA technique depends on either the choice of the anesthesiologist or based on the underlying comorbidities. At our institute, we anesthetize elderly patients who come for fixation of femur fracture with multiple comorbidities using RA techniques as mentioned based on comorbidities and overall general condition.

Methods

In a cohort of 184 elderly patients, we analyzed RA techniques employed over a period of five years in elderly patients admitted with fractures of the proximal femur, its hemodynamic implications and thus attempted to find the suitable RA technique with minimal adverse events after ethics committee approval. We also compared the length of stay in the hospital in relation to RA techniques.

Results

The demographic data was comparable with no significant difference in administering the three RA techniques. SAB, CLE and LSPB was implemented at 33.33%, 35.96%, and 30.7% respectively. Perioperative noradrenaline infusion was a feature in patients who received SAB (p<0/001). The higher number of CLE and LSPB patients had a length of stay of fewer than 48 hours whereas most SAB patients had a length of stay of more than 48 hrs (p<0.001).

Conclusion

Elderly patients with multiple comorbidities should be offered CLE instead of SAB so as to maintain stable hemodynamics. RA technique in an elderly patient with multiple comorbidities should be standardized so as to provide uneventful surgical anesthesia.

## Introduction

Elderly patients with comorbidities who sustain complex proximal femoral fractures (PFF) are often referred to specialized orthopedic surgery centers in view of associated comorbidities and the availability of definitive geriatric protocols [1]. No statistical significance was found in mortality between subarachnoid block (SAB) and general anesthesia techniques employed for hip fractures [2,3]. One of the most striking features of the management of PFF is the wide variation in the use of regional anesthesia (RA) techniques, though even in a well-established tertiary center, controversy and variations exist regarding various options of regional anesthesia (RA). SAB is the commonest form of RA utilized in most hospitals. In our single-center experience, we examined the outcomes related to different RA techniques in elderly patients with PFF. The primary aim was to evaluate the impact of various RA techniques employed, its case-specific distribution for elderly American Society of Anesthesiologists’ physical status (ASA-PS) III and IV patients with PFF’s, and their outcomes in the form of hemodynamic complications. The secondary aim was to evaluate the average length of stay in the hospital, in-hospital mortality on the fifth day (day of discharge) in relation to the RA techniques, specific complication associated with a RA technique, and propose standardization of RA techniques for PFFs.

## Materials and methods

This single-center, observational study was approved by the institutional ethical committee (approved on 22nd November 2018). Medical records (preoperative, anesthesia, and postoperative nursing charts) obtained from the medical record department were examined for patients admitted with PFFs and who underwent a proximal femoral nail (PFN). The study population included 184 adults aged 70 to 97 and categorized in ASA-PS III and IV, between January 2015 to June 2018. Of 184 patients, 114 patients (50 male; 64 female) qualified for the study. In 70 patients, GA was administered in patients on oral anticoagulants or low molecular weight heparins, and inadequate RA qualified for the exclusion.

In our institution, with an INR less than 1.5, an activated partial thromboplastin time (aPTT) less than 35 seconds, platelet count of more than 80,000, last dose of antiplatelet [clopidogrel] stopped for more than five days and last dose of low molecular weight heparin injection [enoxaparin] more than 12 hours are suitable candidates for neuraxial anesthesia. An unusual, prolonged aPTT was screened for deficient coagulation factors by the hematologist.

Based on the available information obtained from the charts the patients were assigned to one particular category of comorbid (example: underwent coronary artery bypass grafting, diabetes mellitus, coronary artery disease). Records were tracked for vital parameters, laboratory investigations, preoperative medications, x-ray chest and spine, electrocardiogram and echocardiography, and another opinion from a specialist consultant if performed.

Information regarding preoperative counseling and patient informed consent was obtained from the anesthesia charts. The type of RA technique employed in each case and the details of the technique were noted. The type of RA implemented in a particular cohort of comorbidities was also noted. On arrival in the operating room (OR) after appropriately sized intravenous (IV) cannulation and application of essential monitors (pulse oximeter, non-invasive blood pressure cuff, electrocardiograph), all patients received ultrasound-guided femoral nerve block with 1% lidocaine 10 ml in the supine position. SAB and continuous lumbar epidural (CLE) were administered in either sitting or lateral position and lumbosacral plexus block (LSPB) in the lateral position with operated side non-dependant.

SAB was administered using 0.5% bupivacaine 2-2.5ml with 150µg buprenorphine as an adjuvant. CLE was in the form of a continuous infusion through a 20 G catheter at the L2-3 level. An initial injection of 8-10ml 0.5% bupivacaine followed by another bolus of 3-5ml of the same solution was administered through the catheter only if the level of surgical anesthesia was not adequate. Postoperatively all patients received 0.1% ropivacaine at 6ml / hour for 48 hours. For LSPB, a compound solution of 0.5% bupivacaine 2mg/kg and 1.5% lidocaine 6mg/kg with 1µg/kg clonidine was used for LSPB achieved with neurostimulation [0.4mg.kg-1for lumbar plexus block (LPB) and 0.2mg/kg for sacral plexus block (SPB)]. Quadriceps contractions at 0.4mA and plantar/dorsiflexion at 0.4mA were the endpoints for LPB and SPB respectively.

All surgeries were performed in the lateral decubitus position with operative site non-dependant without a fracture table, by one of three experienced orthopedic surgeons, practicing for 20 years. The RA techniques were performed by a single anesthesiologist with 25 years of experience in RA. Invasive monitoring in the form of an arterial line and a central venous catheter was performed in patients with low ejection fraction (less than 30%) and in presence of regional wall motion abnormalities. Intravenous paracetamol 1gm was infused at skin closure and every eighth hour thereafter, as a part of multimodal regimen in all groups. IV rescue analgesic was in the form of fentanyl 10μg/ml, initiated at 2ml/hour after a bolus of 0.5μg/kg.

All patients were transferred to the intensive care unit (ICU), and if hemodynamically stable and not requiring monitoring were admitted to the ward. Patients who required perioperative vasoactive drugs (noradrenaline infusion=NAD infusion, dopamine infusion=DOP) were noted and analyzed separately.

The continuous variables were summarised using mean+/- standard deviation or median (interquartile range) as necessary, and categorical variables by percentage or absolute numbers. The difference in mean between groups of types of anesthesia was analyzed by one-way analysis of variation (ANOVA). The association between categorical variables and type of anesthesia was determined by Fisher's exact test. SPSS Statistics v. 21 (IBM Corp., Armonk, NY) was used for statistical analysis. A p-value of less than 0.05 was considered statistically significant.

## Results

Overall, 114 ASA-PS III and IV patients underwent surgery for PFF between January 2015 and June 2018. Of 114 patients, 77 had intertrochanteric fractures while 37 suffered from subtrochanteric fractures. Thirty-eight patients received a SAB, 41 patients a continuous CLE, and 35 patients were administered an LSPB (Table 1).

**Table 1:** Demographic characteristics of participants CLE: continuous lumbar epidural; LSPB: lumbosacral plexus block; SAB: subarachnoid block, ASA-PS: American Society of Anesthesiologists’-Physical Status; IT: intertrochanteric; ST: subtrochanteric Statistical significance: p<0.05

		CLE (n=41)	LSPB (n=35)	SAB (n=38)	p-value
Age in years Mean ± standard deviation		77.39±1.43	77.63±1.64	80.39±1.54	p=0.305
Gender	Male n (%)	18 (36%)	15 (30%)	17 (34%)	p=0.987
	Female n (%)	23 (35.9%)	20 (31.3%)	21 (32.8%)	
Fracture type	IT left n (%)	19 (33.33%)	20 (35.1%)	18 (31.6%)	p=0.6
	IT right n (%)	18 (39.1%)	12 (26.1%)	16 (34.8%)	
	ST left n (%)	2 (33.3%)	3 (50%)	1 (16.7%)	
	ST right n (%)	2 (40%)	0 (0%)	3 (60%)	
ASA-PS	III	17 (28.8%)	18 (30.5%)	24 (40.7%)	p=0.156
	IV	24 (43.6%)	17 (30.9%)	14 (25.5%)	

The median age was 80 years with an interquartile range of (74.5-80.28). In ASA-PS III and IV patients, a CLE was administered more often than LSPB, whereas SAB was utilized the least (p<0.001) (Table 2).

**Table 2:** Association of comorbidities and adverse events with type of anesthesia NAD: noradrenaline; Y: yes; N: no; CNS: central nervous system; CAD: coronary artery disease; CLE: continuous lumbar epidural; LSPB: lumbar and sacral plexus block; SAB: subarachnoid block

		Type of anesthesia			
		CLE (n=41)	LSPB (n=35)	SAB (n=38)	p-value
Cardiovascular	N	5 (45.5%)	5 (45.5%)	1(9.1%)	p=0.163
	Y	36 (35.0%)	30 (29.1%)	37 (35.9%)	
CAD	N	15 (22.4%)	20 (29.9%)	32 (47.8%)	p<0.001
	Y	26 (55.3%	15 (31.9%)	6 (12.8%)	
Respiratory	N	31 (34.1%)	28 (30.8%)	32 (35.2%)	p=0.635
	Y	10 (43.5%)	7 (30.4%)	6 (26.1%)	
Endocrine	N	14 (29.2%)	17 (35.4%)	17 (35.4%)	p=0.419
	Y	27 (40.9%)	18 (27.3%)	21 (31.8%)	
Renal	N	26 (38.2%)	24 (35.3%)	18 (26.5%)	p=0.151
	Y	15 (32.6%)	11 (23.9%)	20 (43.5%)	
CNS	N	28 (36.8%)	22 (28.9%)	26 (34.2%)	p=0.848
	Y	13 (34.2%)	13 (34.2%)	12 (31.6%)	
Malignancy	N	37 (34.3%)	34 (31.5%)	37 (34.3%)	p=0.368
	Y	4 (66.7%)	1 (16.7%)	1 (16.7%)	
Electrolyte	N	13 (27.7%)	14 (29.8%)	20 (42.6%)	p=0.166
	Y	28 (41.85%)	21 (31.3%)	18 (26.9%)	
Spine instrumentation, metastasis	N	41(48.80%)	5(5.95%)	38(45.23%)	p<0.00001
	Y	0(0)	30(100%))	0(0)	
NAD	N	40 (40.4%)	33 (33.3%)	26 (26.3%)	p<0.001
	Y	1 (6.7%)	2 (13.3%)	12 (80%)	
Mortality	N	40 (36.7%)	33 (30.3%)	36 (33%)	p=0.745
	Y	1 (20%)	2 (40%)	2 (40%)	
Cause of mortality	N	40 (36.7%)	33 (30.3%)	36 (33%)	p=0.258
	ACS	1 (50%)	1 (50%)	0 (0%)	
	AMI	0 (0%)	1 (100%)	0 (0%)	
	PE	0 (0%)	0 (0%)	2 (100%)	

The comorbidities were comparable for all three RA approaches except in patients with coronary artery disease (CAD) (p<0.01) who received CLE. In patients with spine pathologies and instrumentation, all received an LSPB (p=0.002735) but considering the sample size, no statistical significance could be derived when compared to the other two techniques (Table 2). Hemodynamic was well preserved in all patients in whom a CLE or an LSPB was administered. In 38 patients who received a SAB (46.15%), NORAD infusion was used intraoperatively and, in the ICU, (p <0.001). The length of stay was significantly associated with the type of anesthesia. A higher number of CLE and LSB patients had a length of stay of 24 hrs and less than 48 hrs, whereas most SAB patients had a length of stay of more than 48 hrs (p<0.001, Table 3). In-hospital mortality was at 2.8% and 5.2% in patients who received LSPB and SAB, and none in whom CLE was administered. The overall mortality stood at 2.63%.

**Table 3:** Association of the length of stay in ICU with the type of anesthesia CLE: continuous lumbar epidural; LSPB: lumbar and sacral plexus block; SAB: subarachnoid block

Length of stay	Type of anesthesia			p-value
	CLE (n=41)	LSPB (n=35)	SAB (n=38)	
24 hr	24(54.5%)	20 (45.5%)	0 (0.0%)	<0.00001
48 hr	12 (28.6%)	9 (21.4%)	21 (50.0%)	
72 hr	0 (0.0%)	0 (0.0%	10 (100.0%)	
96 hr	3 (23.1%)	4 (30.8%	6 (46.2%)	
120 hr	2 (40.0%)	2 40.0%	1 (20.0%)	

## Discussion

Our aim was to understand the impact of different types of RA techniques implemented in PFF undergoing a PFN, and outcomes in the form of whether there was an inclination of utilizing these techniques in a particular cohort with comorbidities and hemodynamic complications. Epidural was consistently effective in all ASA-PS III and IV, for anesthesia and analgesia purposes, and patients were hemodynamically stable throughout the perioperative course in patients with CAD. LSPB was administered particularly in the operated spine and metastatic spine disorders. SAB was associated with increased use of IV vasoactive drugs in the postoperative period.

Elderly patients with comorbidities sustaining complex PFFs require an early optimization and surgical intervention. Compared to general anesthesia, RA is associated with less incidence of in-hospital mortality and respiratory-related morbidity [4]. The type of a safe and feasible RA remains undefined and inconclusive in a cohort of PFFs. SAB was the only technique that is described, but discussion on other options like CLE and LSPB remains elusive. The Anesthesia Sprint Audit of Practice (ASAP) reports that the conduct of anesthesia appears to affect outcomes after hip fracture surgery [5]. With a mean arterial blood pressure (ABP) of less than 55mmHg for more than 20 minutes, a graded increase in the risk of renal and myocardial injury was noted [6]. In spite of these problems, anaesthesiologists continue to use SAB for PFFs without understanding the altered pathophysiology it creates in highly comorbid and frail patients [7]. Based on our study with a limited sample size, we recommend avoiding SAB in elderly patients with cardiac ailments (EF less than 30%, presence of regional wall motion abnormality in echocardiography, long-standing diabetes with cardiomyopathy). The CLE is associated with a limited sympathectomy as against the extensive sympathectomy which is characteristic of SAB. We recommend incremental epidural anesthesia to have stable hemodynamic in the perioperative period. Further LA top-ups in epidural space allow extension of surgical anesthesia and postoperative pain relief through infusion pumps. Improved outcomes are directly related to the use of CLE [8].

Neuraxial techniques are challenging in patients with operated spine instrumentation [9]. Further neurological adverse events are known in patients with metastatic spine undergoing a SAB [10]. Not everyone is trained to deliver high-quality anesthesia using advanced nerve blocks in geriatric patients with PFFs. The LSPB are advanced deep blocks [11-13]. The risk profile of LPB is high as it could lead to epidural diffusion, potential local anesthesia toxicity, and lumbar plexopathy [14]. In view of advanced block and high-risk profiles, we recommend that LSPBs should be performed by experts. Although ultrasound provides a predictable paravertebral sonoanatomy for lumbar plexus and of sacral plexus blocks in adults, the same is not true in elderly patients [15]. A study by de Visme et al. mentions hypotension after SAB in patients over the age of 85 years was of larger magnitude and sustained for a long time [16]. Invasive cardiac monitoring using arterial and central line during an LSPB for hip surgery observed a significant drop in the MAP though there was no change in the cardiac index, ABP, and heart rate [17]. The in-hospital mortality was selected as the secondary study endpoint because of its probable immediate co-relation in the postoperative period. The 30th day and one-year mortality are never affected by the type of anesthesia used during surgery. However, in our series, RA techniques were employed in ASA-PS III and IV patients, and there was no mortality in patients who received CLE but it was 2.8% and 5.2% of patients who were administered LSPB and SAB respectively, with overall mortality at 2.63%. Incremental epidural analgesia can be achieved with titrated doses of local anesthetic injected in the lumbar epidural space. In our cases who received segmental CLE, patients with severe CAD and respiratory disorders had better outcomes.

The sample size was a major limitation. Confounders (patients were heterogeneous with multiple comorbidities present in the same patient) and selection bias are limitations in an observational study; however, a randomized controlled trial is unfeasible for a similar study. Interventions in the observational studies are determined by clinical practice and not a protocol [18].

## Conclusions

Elderly patients with significant comorbidities should be offered CLE instead of SAB so as to maintain stable hemodynamics. RA technique in elderly patients with multiple comorbidities should be standardized so as to provide uneventful surgical anesthesia. Elderly patients undergoing the surgical procedure are susceptible to postoperative cognitive dysfunction and delirium, secondary to higher pain scores and opioid levels. However, continuous epidural analgesia benefits in preventing such occurrences, leading to an early hospital discharge. We recommend CLE anesthesia in elderly patients with ASA-PS III and IV. An LSPB should be preferred in instrumented and pathological spines. SAB should be sparingly administered in these patients given the unstable perioperative hemodynamics and an increased ICU stay.
